# Forkhead box O3 promotes colon cancer proliferation and drug resistance by activating MDR1 expression

**DOI:** 10.1002/mgg3.554

**Published:** 2019-01-08

**Authors:** Zhuanglei Gao, Zhaoxia Li, Yuelin Liu, Zhonghao Liu

**Affiliations:** ^1^ Department of Gastrointestinal Surgery The Second Hospital of Shandong University Jinan China; ^2^ Department of Pediatrics The Second Hospital of Shandong University Jinan China; ^3^ Department of Surgery Bincheng District Municipal Hospital, Bincheng District Binzhou China; ^4^ Department of Trauma and Orthopaedics The Second Hospital of Shandong University Jinan China

**Keywords:** colon cancer, doxorubicin resistance, FOXO3, MDR1, proliferation

## Abstract

**Background:**

Globally, colon cancer (CC) is the third reason of tumor‐related deaths. Previous reports indicate that Forkhead box O3 (FOXO3) is involved in the development of various tumors and may have different effects depending upon the types of tumors. Hence, this study was to examine the effects of FOXO3 on CC cells and uncover the possible mechanisms.

**Methods:**

MTT and cell count assay were applied to analyze the viability of transfected CC cells. rVista, dual luciferase reporter assay, and chromatin immunoprecipitation assay were used to identify the downstream target of FOXO3 in HCT116 cells. The mRNA and protein abundance of FOXO3 and MDR1 were determined by quantitative PCR and Western blot, respectively.

**Results:**

Forkhead box O3 stimulated the proliferation of both HCT116 and DLD1 cells. Moreover, FOXO3 overexpression inhibited doxorubicin sensitivity of HCT116 cells, while the knockout of FOXO3 by FOXO3 shRNA restored the doxorubicin sensitivity in doxorubicin‐resistant HCT116 DR cells. Next, we found that FOXO3 directly bound to the promoter of MDR1 and enhanced MDR1 expression in HCT116 cells. MDR1 overexpression enhanced the viability and doxorubicin resistance of CC cells. Besides, MDR1 overexpression plasmid significantly abrogated the decrease in cell proliferation and resistance of HCT116 cells to doxorubicin caused by FOXO3 knockout.

**Conclusion:**

Forkhead box O3 exhibited promotive effects on the proliferation and doxorubicin resistance in CC cells via targeting MDR1.

## 1 INTRODUCTION

Colon cancer (CC), also known as colorectal or bowel cancer, is the third most frequent malignant cancer, and more than 65 percent of CC cases are found in developed countries (Kotaka et al., 2018). Aging is the most critical risk factor for CC, and other risk factors for CC include obesity, smoking, drinking, lack of physical activity, inflammatory bowel diseases, and inherited genetic disorders (Board, 2002; Theodoratou, Timofeeva, Li, Meng, & Ioannidis, 2017). CC limited to the colon wall may be cured by surgical resection, whereas there is no curative treatment for CC patients present with metastatic disease. Chemotherapy is often used for clinical CC treatment, whereas drug resistance is a frequent occurrence. Therefore, there are urgent needs for investigating the underlying mechanisms of CC progression and chemotherapy resistance to develop new potent therapies for CC treatment.

Forkhead box O3 (FOXO3), also known as FOXO3a, is one of FOXO subfamily members (Bullock et al., 2013). Emerging evidence indicates that FOXO3, characterized by a “Forkhead box” DNA‐binding domain, plays important roles in carcinogenesis and chemotherapeutic resistance (Gomes, Zhao, & Lam, 2013). For example, Yang et al. (2010) showed that inhibited FOXO3 expression and activation enhanced the resistance to AZD6244 treatment in cells of various tumors. Wei et al. (2016) demonstrated that impaired FOXO3 expression contributed to the chemoresistance of ovarian cancer cells.

Due to the previous findings, we wonder whether FOXO3 is involved in CC progress and drug resistance or not. Hence, we investigated the biological effects of FOXO3 on the proliferation and drug resistance of human CC cells and explored the possible mechanisms.

## 2 METHODS AND MATERIALS

### 2.1 Cell lines

HCT116 and DLD1 human cells were bought from ATCC. These cells were cultured in McCoy's 5A media (Sigma, St. Louis, MO, USA) supplemented with 10% fetal bovine serum (Gibco, Grand Island, NY, USA) at 37°C in 5% CO_2_. Doxorubicin‐resistant HCT116 DR cells were established as previously described (Choi, Kim, Choi, Kim, & Lee, 1996).

### 2.2 Plasmid construction and transfection

Scramble shRNA and FOXO3 shRNA were synthesized and cloned into LV3 vector (Sangon, Shanghai). FOXO3 and MDR1 were amplified and subcloned into MSCV‐PIG vector to construct FOXO3 overexpression plasmid and MDR1 overexpression plasmid, respectively. Cell transfection was conducted through Lipofectamine 2000 (Invitrogen, Carlsbad, CA, USA).

### 2.3 Western blot analysis

Primary antibodies for FOXO3 and MDR1 were obtained from Cell Signaling. Cells were harvested and lysed in RIPA (Beytime, Shanghai, China). Equal amount of proteins from each group was subjected to 10% SDS‐PAGE and transferred to nitrocellulose membrane. Prepared blots were blocked by TBS containing 3% BSA. Subsequently, the membrane was incubated with the specific primary antibodies (1:500 or 1:1,000 dilution) followed by HRP‐linked secondary antibodies.

### 2.4 Dual luciferase reporter assay

Wild‐type MDR1 promoter harboring potential FOXO3 binding sites was amplified and subcloned into pGL3 basic vector (Promega, Madison, WI, USA) to construct pGL3‐MDR1‐WT‐promoter plasmid. The mutant MDR1 promoter harboring the mutant FOXO3 binding sequences was amplified to construct pGL3‐MDR1‐mut‐promoter plasmid. HCT116 cells were co‐transfected with different reporter vectors (pGL3 basic vector, pGL3‐MDR1‐WT‐promoter, or pGL3‐MDR1‐mut‐promoter) as well as FOXO3 overexpression plasmid or the empty vector. At 48 hr, luciferase activity was measured via the dual luciferase assay system (Promega).

### 2.5 MTT assay

Colon cancer cells (10^3 ^cells/well) were inoculated into 96‐well plates, and 0.5 mg/ml MTT solution was added into each well for 4 hr. Then, the solution was discarded and replaced with 150 µl of DMSO to lyse the crystalline precipitate. Absorbance at 545 nm was detected using a microplate reader.

### 2.6 Quantitative PCR (qPCR) analysis

Quantitative PCR was conducted with 0.5 μg total RNA using the ABI 7500 System (Life Technologies, NY, USA). Primer sequences were listed below:

FOXO3 fwd, 5′‐CGGACAAACGGCTCACTCT‐3′;

FOXO3 rev, 5′‐GGACCCGCATGAATCGACTAT‐3′;

MDR1 fwd, 5′‐TTGGCTGATGTTTGTGGGAAG‐3′;

MDR1 rev, 5′‐CCAAAAATGAGTAGCACGCCT‐3′.

### 2.7 Chromatin immunoprecipitation (ChIP) assay

Chromatin immunoprecipitation experiment was performed according to the previous paper (Wu et al., 2015) to examine the binding between FOXO3 and MDR1 promoter. In brief, cells were fixed, lysed and sonicated. After centrifugation, soluble chromatin fragments were pretreated with protein G‐agarose and subsequently incubated with either FOXO3 antibody (Cell Signaling) or rabbit IgG (Cell Signaling) overnight at 4°C. Extracted DNA was analyzed by PCR. The primers for ChIP assay were as follows:

MDR1 promoter:

Forward: 5′‐TACACCTCTTTAGGGTTAAGGCA‐3′

Reverse: 5′‐GGACGTGAAGATAGACAACTGGT‐3′.

### 2.8 Statistical analysis

Data were generated from at least three separate tests. SPSS 10.0 software was employed for statistical analysis. In cases of statistical significance, the ranked parameters were compared by one‐way ANOVA analysis or Student's *t* test. *p* < 0.05 indicated significance.

## 3 RESULTS

### 3.1 FOXO3 promoted the proliferation of HCT116 and DLD1 cells

**Figure 1:**
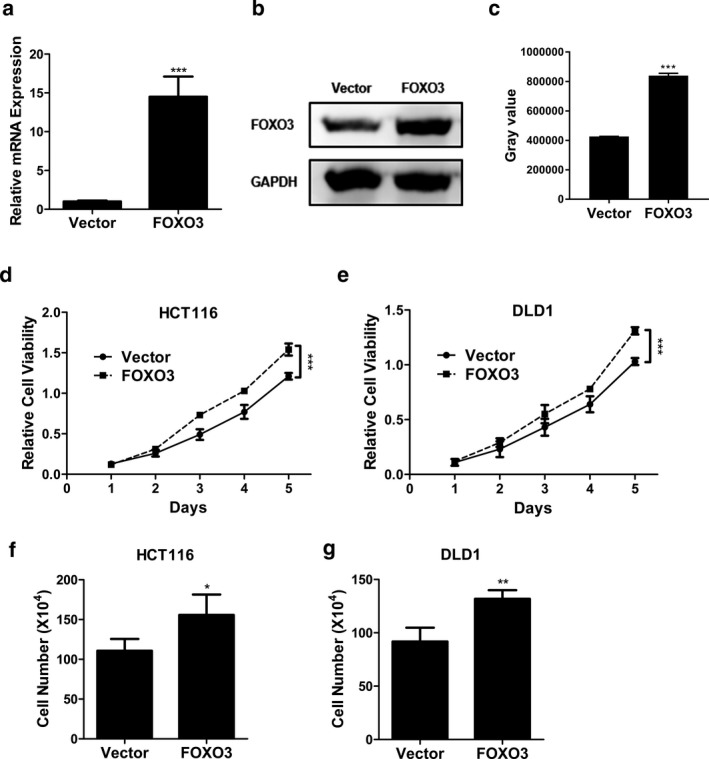
FOXO3 promoted CC cell proliferation. (a, b) The mRNA (a) and protein expression (b) of FOXO3 in HCT116 cells transfected with FOXO3 overexpression plasmid (FOXO3) or empty vector (Vector) were determined by qPCR and Western blot, respectively. (c) The gray scale analysis of the Western blot in (b). (d) Cell viability of HCT116 cells transfected with FOXO3 overexpression plasmid (FOXO3) or empty vector (Vector) was determined by MTT assay. (e) Cell viability of DLD1 cells transfected with FOXO3 overexpression plasmid (FOXO3) or empty vector (Vector) was determined by MTT assay. (f) Cell viability of HCT116 cells transfected with FOXO3 overexpression plasmid (FOXO3) or empty vector (Vector) was determined by cell count assay. (g) Cell viability of DLD1 cells transfected with FOXO3 overexpression plasmid (FOXO3) or empty vector (Vector) was determined by cell count assay. Data were shown as mean ± *SD* *p < 0.05; **p < 0.01; ***p < 0.001 (ANOVA test in figure (d) and (e), others Student's *t* test)

In current study, HCT116 and DLD1 cells were transfected with FOXO3 overexpression plasmid (FOXO3) or empty vector (Vector). qPCR and Western blot assay confirmed that FOXO3 mRNA (Figure 1a, *p* < 0.001) and protein levels (Figure 1b,c, *p* < 0.001) in HCT116 cells in FOXO3 group were much higher than those in the vector group. Figure 1d,e indicated that the viability of HCT116 and DLD1 cells was promoted by FOXO3 overexpression in a time‐dependent manner, compared with those in the vector groups, respectively (*p* < 0.001). In addition, cell count assay also showed that compared with those in the matched vector groups, FOXO3 overexpression enhanced HCT116 and DLD1 cell proliferation by 35.9% and 42.2%, respectively (Figure1f,g, *p* < 0.05 and *p* < 0.01, respectively).

### 3.2 FOXO3 decreased doxorubicin sensitivity in doxorubicin‐resistant colon cancer cells

**Figure 2:**
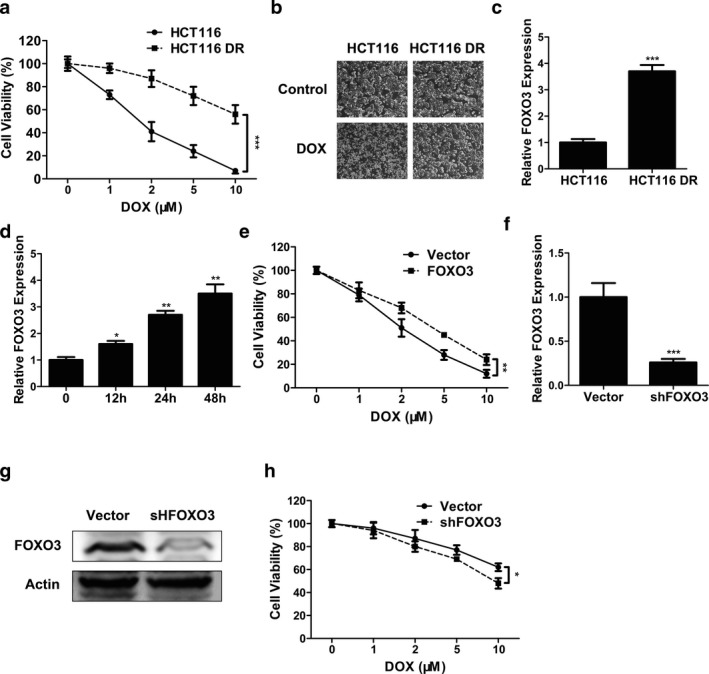
FOXO3 decreased doxorubicin sensitivity in doxorubicin‐resistant colon cancer cells. (a) Cell viability of HCT116 and doxorubicin‐resistant HCT116 DR cells treated with different concentration of doxorubicin was determined by MTT assay. (b) The representative images of HCT116 and HCT116 DR cells treated with or without DOX. (c) The expression levels of FOXO3 in HCT116 and HCT116 DR cells were determined by qPCR. (d) The expression levels of FOXO3 in HCT116 cells treated with 4 μM doxorubicin for different times were determined by qPCR. (e) Cell viability of HCT116 cells transfected with FOXO3 overexpression plasmid (FOXO3) or empty vector (Vector) and treated with different concentration of doxorubicin was determined by MTT assay. (f, g) The expression levels of FOXO3 in HCT116 DR cells transfected with FOXO3 shRNA plasmid (shFOX3) or empty vector (Vector) were determined by qPCR (f) or Western blot (g). (h) Cell viability of HCT116 DR cells transfected with FOXO3 shRNA plasmid (shFOX3) or empty vector (Vector) and treated with different concentration of doxorubicin was determined by MTT assay. Data were shown as mean ± *SD* *p < 0.05; **p < 0.01; ***p < 0.001 (ANOVA test in figure (a), (c), (d) and (g), others Student's *t* test)

To investigate the possible connection between FOXO3 and doxorubicin (DOX) sensitivity of CC cells, HCT116 and HCT116 DR cells were treated with different concentration of doxorubicin (0, 1, 2, 5, 10 μm). At 24 hr, MTT assay demonstrated that doxorubicin reduced the proliferation of HCT116 and HCT116 DR cells in a dose‐dependent fashion. Moreover, HCT116 DR cells were more resistant to doxorubicin than HCT116 cells, as shown by the increased viability when exposed to the same concentration of doxorubicin (Figure 2a,b, *p* < 0.001). Figure 2c revealed that HCT116 cells had lower FOXO3 mRNA level than HCT116 DR cells (*p* < 0.001). Moreover, Figure 2d also indicated that doxorubicin treatment time‐dependently upregulated FOXO3 mRNA expression in HCT116 cells (*p* < 0.05, *p* < 0.01 and *p* < 0.01 for 12, 24 and 48 hr, respectively).

Forkhead box O3 overexpression plasmid enhanced the resistance of HCT116 cells to doxorubicin compared to empty vector (Figure 2e, *p* < 0.01). On the other hand, FOXO3 shRNA plasmid inhibited FOXO3 mRNA (Figure 2f, *p* < 0.001) and protein expression (Figure 2g) in HCT116 DR cells. Moreover, FOXO3 shRNA reduced the doxorubicin resistance of HCT116 DR cells (Figure 2h, *p* < 0.05).

### 3.3 MDR1 is a direct downstream target of FOXO3

**Figure 3:**
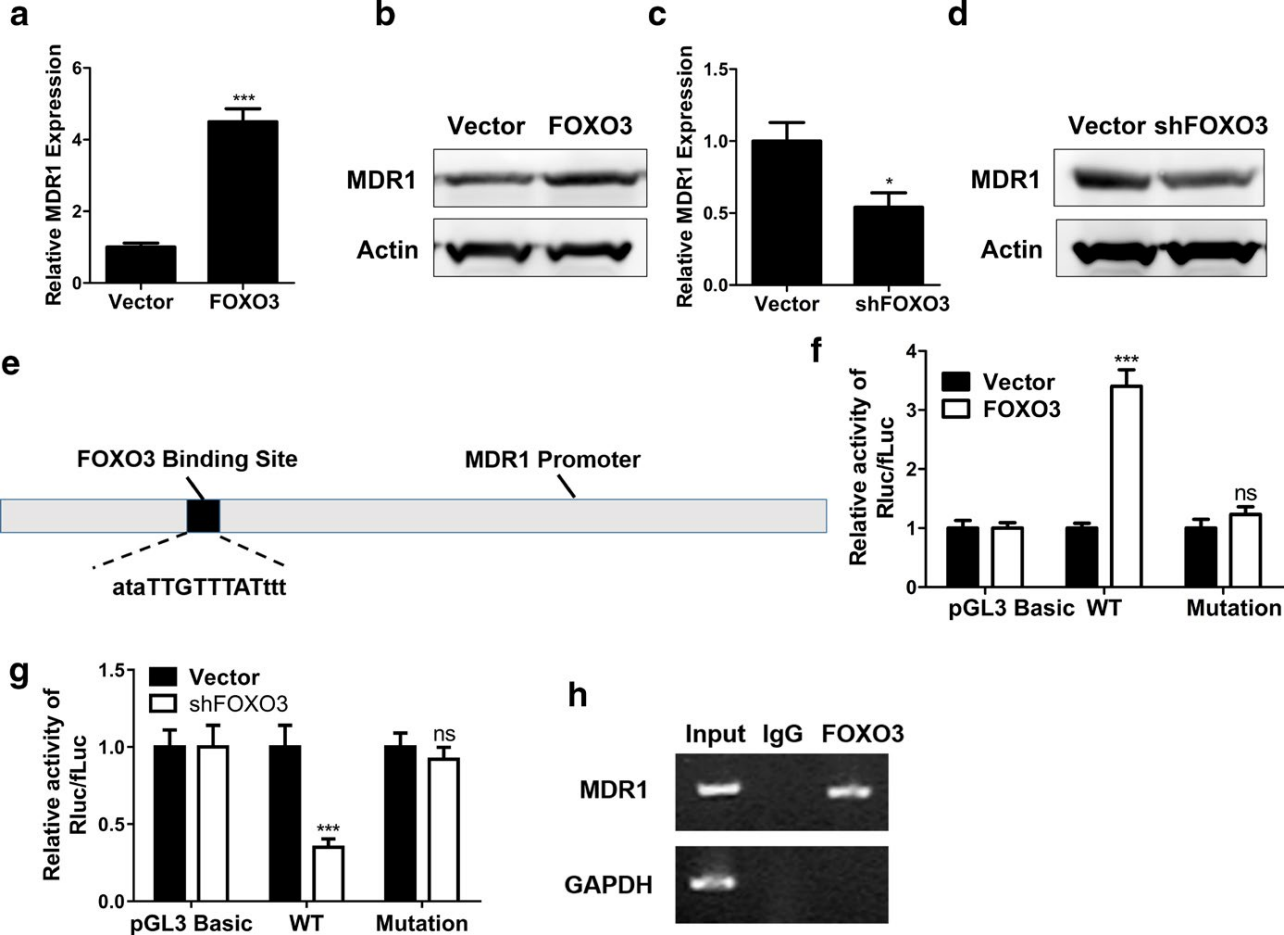
FOXO3 activated MDR1 expression. (a, b) The expression of MDR1 in HCT116 cells transfected with empty vector (Vector) or FOXO3 overexpression plasmid (FOXO3) was determined by qPCR (a) or Western blot (b). (c, d) The expression of MDR1 in HCT116 cells transfected with empty vector (Vector) or FOXO3 shRNA plasmid (shFOX3) was determined by qPCR (c) or Western blot (d). (e) Schematic diagram of FOXO3 binding site on the promoter of MDR1. (f) Dual luciferase reporter assay for MDR1 promoter (wild‐type or mutant) activity. The promoter constructs were co‐transfected with empty vector (Vector) or FOXO3 overexpression plasmid (FOXO3). (g) Dual luciferase reporter assay for MDR1 promoter (wild‐type or mutant) activity. The promoter constructs were co‐transfected with empty vector (Vector) or FOXO3 shRNA (shFOXO3). (h) CHIP assay of the FOXO3 antibody or IgG negative control. The enrichment of MDR1 promoter was determined by PCR, GAPDH promoter served as negative control. Data were shown as mean ± *SD* *p < 0.05; ***p < 0.001; ns, not significant (Student's *t* test)

Forkhead box O3 overexpression stimulated MDR1 mRNA (Figure 3a, *p* < 0.001) and protein expression (Figure 3b) in HCT116 cells, compared to the control group. In contrast, HCT116 cells transfected with FOXO3 shRNA plasmid had lower MDR1 mRNA (Figure 3c, *p* < 0.05) and protein levels (Figure 3d) compared with cells transfected with empty vector. Potential targets of FOXO3 were predicted by rVista analysis, and Figure 3e demonstrated that there was a potential FOXO3 binding site in MDR1 promoter. Figure 3f revealed that FOXO3 overexpression plasmid enhanced the luciferase activity of the reporter plasmid containing wide‐type MDR1 promoter compared with the empty vector (*p* < 0.001), whereas it had no significant effect on the luciferase activity of the reporter plasmid containing mutant MDR1 promoter. In addition, FOXO3 shRNA inhibited the luciferase activity of the reporter plasmid containing wide‐type MDR1 promoter compared with control (*p* < 0.001), whereas FOXO3 shRNA had no significant effect on the luciferase activity of the reporter plasmid containing mutant MDR1 promoter (Figure 3g). Besides, CHIP assay further proved that FOXO3 directly bind to the seed region in MDR1 promoter (Figure 3h).

### 3.4 MDR1 had tumor‐promoting effects on the proliferation and drug resistance of CC cells

**Figure 4:**
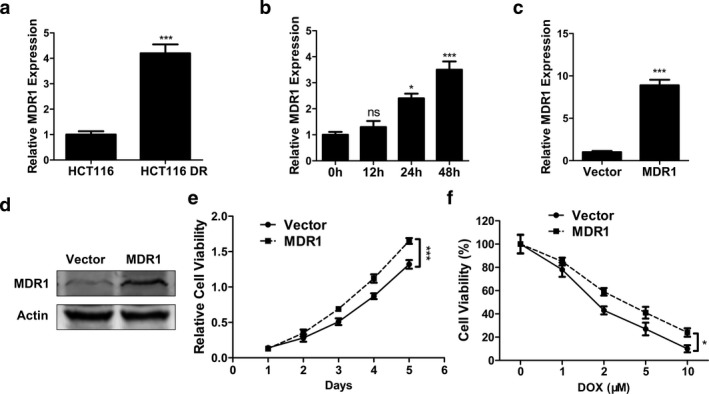
MDR1 exhibited prompting effect on CC cell proliferation and drug resistance. (a) The expression levels of MDR1 in HCT116 and HCT116 DR cells were determined by qPCR. (b) The expression levels of MDR1 in HCT116 cells treated with 4 μM doxorubicin for different times were determined by qPCR. (c, d) The expression levels of MDR1 in HCT116 cells transfected with MDR1 overexpression plasmid (MDR1) or empty vector (Vector) were determined by qPCR (c) or Western blot (d). (e) Cell viability of HCT116 cells transfected with MDR1 expression plasmid (MDR1) or empty vector (Vector) was determined by MTT assay. (f) Cell viability of HCT116 cells transfected with MDR1 overexpression plasmid (MDR1) or empty vector (Vector) and with different concentration of doxorubicin was determined by MTT assay. Data were shown as mean ± *SD* *p < 0.05; ***p < 0.001; ns, not significant (ANOVA test in figure (b), (e) and (f), others Student's *t* test)

Quantitative PCR results showed that HCT116 DR cells had higher MDR1 mRNA level than HCT116 cells (Figure 4a, *p* < 0.001). On the other hand, 4 μM doxorubicin time‐dependently upregulated MDR1 expression in HCT116 cells (Figure 4b, *p* < 0.05 and *p* < 0.001 for 24 and 48 hr, respectively). Thus, we speculated that there was a positive relationship between MDR1 expression and the proliferation and drug resistance of CC cells. To test the above hypothesis, HCT116 cells were transfected with MDR1 overexpression plasmid or empty vector. Figure 4c,d showed that HCT116 cells transfected with MDR1 overexpression plasmid had higher MDR1 mRNA (*p* < 0.001) and protein levels than those transfected with empty vector. Next, MTT assay demonstrated that MDR1 overexpression plasmid improved HCT116 cell proliferation in a time‐dependent manner (Figure 4e, *p* < 0.001). On the other hand, MDR1 overexpression enhanced the doxorubicin resistance of HCT116 cells, as shown by the increased cell viability in MDR1 overexpression plasmid group compared to that of empty vector group at the same concentration of doxorubicin (Figure 4f, *p* < 0.05).

### 3.5 MDR1 overexpression blocked the anti‐tumor effects of FOXO3 shRNA on the proliferation and drug resistance of CC cells

**Figure 5:**
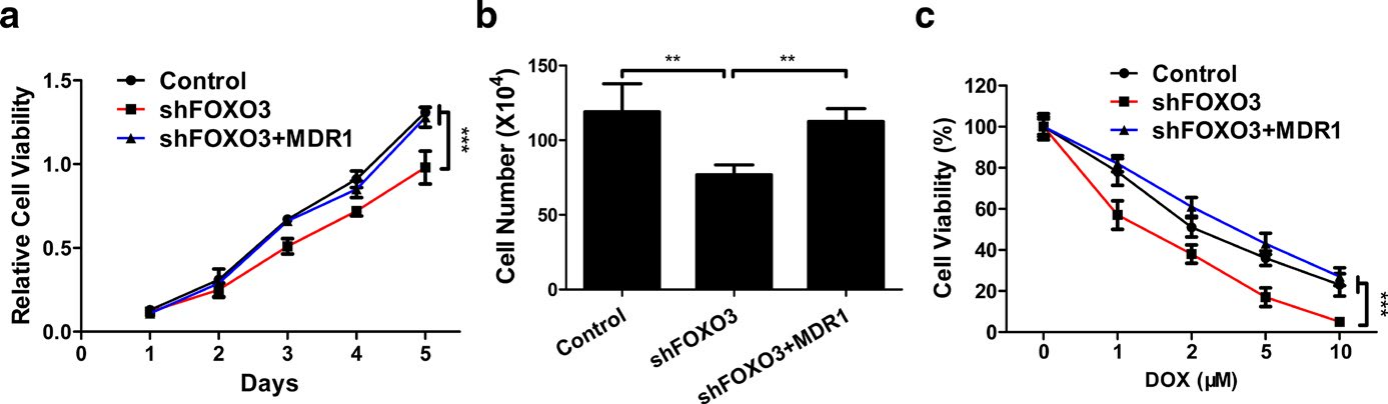
MDR1 was the functional downstream effector of FOXO3. HCT116 cells were transfected with empty vector (control), FOXO3 shRNA plasmid (shFOXO3) or FOXO3 shRNA plasmid plus MDR1 overexpression plasmid (shFOXO3+MDR1). (a, b) Cell viability of transfected HCT116 cells was determined by MTT assay (a) or cell count assay (b). (c) HCT116 cells, transfected with control, shFOXO3 or shFOXO3+MDR1, were treated with different concentration of doxorubicin and cell viability was determined by MTT assay. Data were shown as mean ± *SD* **p < 0.01; ***p < 0.001 (ANOVA test)

Figure 5a showed that MDR1 overexpression plasmid completely restored the decreased viability of HCT116 cells caused by FOXO3 shRNA (*p* < 0.001). Cell count assay also showed that FOXO3 knockout by FOXO3 shRNA decreased the viability of HCT116 cells by 33.33% compared to the control group (Figure 5b, *p* < 0.01). However, MDR1 overexpression plasmid almost completely abolished the above effect of FOXO3 shRNA (Figure 5b, *p* < 0.01). On the other hand, MDR1 overexpression also blocked the inhibitive effect of FOXO3 shRNA on the drug resistance of HCT116 cells (Figure 5c, *p* < 0.001).

## 4 DISCUSSION

The current treatments for CC include surgery, radiation therapy, chemotherapy, and targeted therapy (Li, Shen, Zhou, & Yu, 2018). People with early‐stage CC may be cured via surgery, whereas CC patients at the advanced stages treated with the combined chemotherapy are usually not curable due to drug resistance (Ma et al., 2012; Yu et al., 2017). Increasing evidence suggests that FOXO3 played important roles in CC development and metastasis (Bullock et al., 2013; Tenbaum et al., 2012). Hence, we want to explore the possible relationship between FOXO3 and the proliferation and drug resistance of CC cells to obtain better clinical therapy for advanced CC patients. To our delight, the results of MTT and cell count assay indicated that FOXO3 overexpression fostered the proliferation of HCT116 and DLD1 cells in a time‐dependent fashion.

At present, the application of doxorubicin, a DNA‐intercalating anthracycline antibiotic, in combination with other anti‐tumor drugs exhibits good therapeutic effects against late‐stage CC (Qu et al., 2015). However, chemoresistance is a frequent event and inhibits the clinical application of chemotherapy drugs. Hence, a better understanding about the molecular mechanisms underlying the CC cell resistance to chemotherapy drugs will be very crucial for chemotherapy. In this study, HCT116 DR cells had higher mRNA level of FOXO3 compared to their parental cells. Moreover, doxorubicin time‐dependently stimulated FOXO3 expression in HCT116 cells, which suggested that the upregulation of FOXO3 was a consistent step during the conversion process from initial doxorubicin sensitivity to doxorubicin resistance in CC cells. In addition, FOXO3 overexpression enhanced the resistance of HCT116 cells to doxorubicin, whereas FOXO3 downregulation inhibited the doxorubicin resistance of HCT116 DR cells. Taken together, FOXO3 may positively mediate the doxorubicin resistance of HCT116 cells.

It is generally accepted that activated FOXO3, one of the FOXO transcription factors, accumulates in the nucleus and then bind to DNA or other transcriptional factors to modulate the expressions of its specific target genes related to cell proliferation, apoptosis, and other critical cellular processes (Burgering & Kops, 2002; Myatt & Lam, 2007). To identify the novel targets of FOXO3 in CC cells, rVista analysis and dual luciferase reporter assay were carried out. The results showed that FOXO3 overexpression enhanced the luciferase activity of pGL3‐MDR1‐WT‐promoter plasmid, whereas it had little effect on the luciferase activity of pGL3‐MDR1‐mut‐promoter plasmid, which suggested that FOXO3 may be able to bind to the seed region in MDR1 promoter and stimulated MDR1 expression. Moreover, FOXO3 overexpression significantly stimulated MDR1 expression, whereas FOXO3 shRNA had the opposite effect. In addition, CHIP assay further demonstrated that there was direct binding between FOXO3 protein and MDR1 promoter in HCT116 cells. Hence, these above results suggested that FOXO3 was a binding partner of MDR1 promoter and positively regulated MDR1 expression in HCT116 cells.

MDR1, also known as P‐gp, ABCB1, or CD243, belongs to ATP‐binding cassette family and pumps various foreign substances across the cell membrane. Recent studies found that the MDR1 had important functions in the drug resistant of various tumors. For example, Pan, Miao, and Chen (2018) demonstrated that germacrone inhibited MDR1 expression and subsequently decreased the adriamycin resistance of chronic myelogenous leukemia cells. Zhu, Lv, Yan, and Gao (2013) showed that the knockdown of MDR1 enhanced the adriamycin sensitivity of drug‐resistant gastric cancer cell line SGC7901‐MDR1. Besse et al. (2018) found that MDR1 overexpression was the most important change in carfilzomib‐resistant multiple myeloma cells. Moreover, many studies proved that there was positive relationship between MDR1 and the resistance to various drugs in CC treatment, such as doxorubicin (Du et al., 2018; Yan, Zhao, & Zhang, 2017), oxaliplatin (Zhou et al., 2017), and irinotecan (Paule et al., 2010). In this study, qPCR data further demonstrated that FOXO3 overexpression had little effects on the expression of other five members of ATP‐binding cassette family in HCT116 cells, including ABCB2, ABCA1, ABCA2, ABCG1, and ABCG2 (Figure S1). Moreover, we found that MDR1 overexpression completely restored FOXO3 knockout induced the decrease in the proliferation and doxorubicin resistance of HCT116 cells. Hence, our data suggested that MDR1 was a functional downstream effector of FOXO3 in CC cells.

It is well‐known that the roles of FOXO3 in cancer progress are complicated. Previous studies demonstrated that FOXO3 exhibited suppressive effects on various tumors. For instance, Park et al. (2016) found that FOXO3 activation restricted triple‐negative breast cancer (TNBC) cell survival and proliferation. Wang et al. (2015) showed that FOXO3 contributed to rhein‐stimulated Bim expression and apoptosis in both MCF7 breast cancer cells and HepG2 hepatoma cells. In contrast, more and more studies described the tumor‐promoting actions of FOXO3. For example, Zhang et al. (2016) found that activated FOXO3 negatively modulated the expression of metastasis suppressor gene nm23‐H1 in A549 non‐small‐cell lung cancer cells. Tenbaum et al. (2012) showed that FOXO3 may foster CC metastasis via co‐modulation of metastasis‐related genes. Based on our above findings, we here found that FOXO3 promoted the proliferation and drug resistance of CC cells by activating MDR1 expression.

In summary, our present study characterized that FOXO3 can directly bind to MDR1 promoter and enhanced MDR1 expression, which subsequently fostered the proliferation and doxorubicin resistant of human CC cells. Therefore, our finding suggested that FOXO3 may be a potential therapeutic target during CC treatment.

## 5 CONCLUSION

Forkhead box O3 has a tumor‐promoting role in CC cells via targeting MDR1.

## CONFLICT OF INTEREST

The authors declare that they have no conflict of interest.
