# Supplementary material for: Forkhead box O3 promotes colon cancer proliferation and drug resistance by activating MDR1 expression
Source: Mol Genet Genomic Med. 2019 Jan 8;7(3):e554. doi: 10.1002/mgg3.554 (PMC6418361; doi:10.1002/mgg3.554)
Supplement: Supplementary file 1 [file MGG3-7-na-s001.zip › mgg3554-sup-0001-FigS1/mgg3554-sup-0001-FigS1.docx]

**Supplementary Materials**


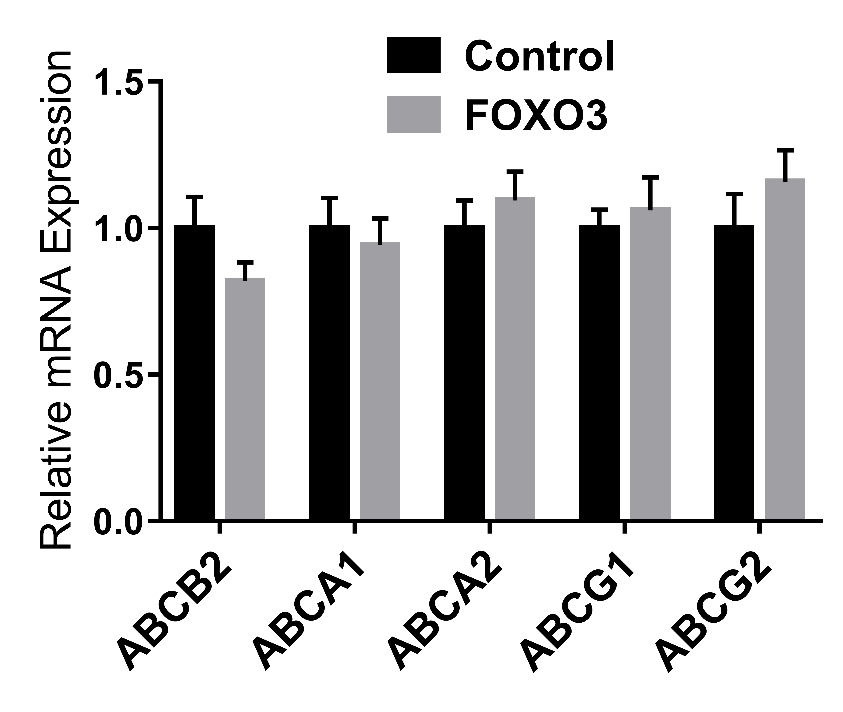


Figure S1. A panel of ATP binding cassette subfamily in HCT116 cells transfected with FOXO3 was determined by qPCR.
