# Supplementary figures and images for: Forkhead box O3 promotes colon cancer proliferation and drug resistance by activating MDR1 expression
Source: Mol Genet Genomic Med. 2019 Jan 8;7(3):e554. doi: 10.1002/mgg3.554 (PMC6418361; doi:10.1002/mgg3.554)

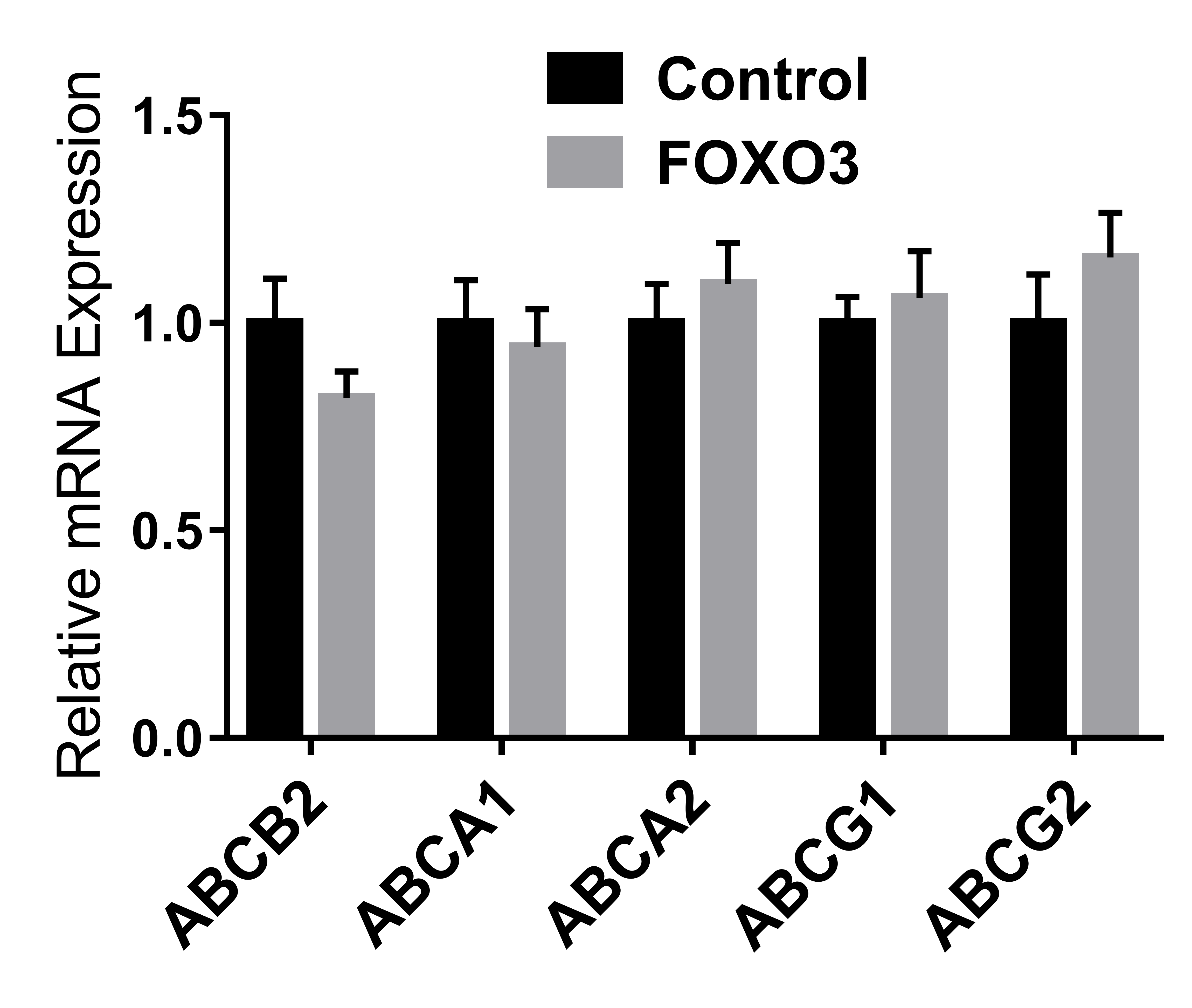

Supplement: Supplementary file 1 [file MGG3-7-na-s001.zip › mgg3554-sup-0001-FigS1/mgg3554-sup-0001-FigS1.tif]
